# Prevalence and Outcomes of Major Psychiatric Disorders Preceding Index Surgery for Degenerative Thoracic/Lumbar Spine Disease

**DOI:** 10.3390/ijerph18105391

**Published:** 2021-05-18

**Authors:** Yu-Chi Huang, Chih-Hui Chang, Chih-Lung Lin, Liang-Jen Wang, Chih-Wei Hsu, Yu-Feng Su, Yi-Ching Lo, Chi-Fa Hung, Yun-Yu Hsieh, Cheng-Sheng Chen

**Affiliations:** 1 Graduate Institute of Clinical Medicine, College of Medicine, Kaohsiung Medical University, Kaohsiung 807, Taiwan; ychuang01@gmail.com; 2 Department of Psychiatry, Kaohsiung Chang Gung Memorial Hospital, College of Medicine, Chang Gung University, Kaohsiung 833, Taiwan; harwicacademia@gmail.com (C.-W.H.); chifa.hung@gmail.com (C.-F.H.); 3 Department of Surgery, Division of Neurosurgery, Kaohsiung Medical University Hospital, College of Medicine, Kaohsiung Medical University, Kaohsiung 807, Taiwan; chchang20@gmail.com (C.-H.C.); chihlung1@yahoo.com (C.-L.L.); suyufeng2000@gmail.com (Y.-F.S.); 4 Department of Child and Adolescent Psychiatry, Kaohsiung Chang Gung Memorial Hospital, College of Medicine, Chang Gung University, Kaohsiung 833, Taiwan; wangliangjen@gmail.com; 5 Department of Pharmacology, College of Medicine, Kaohsiung Medical University, Kaohsiung 807, Taiwan; yichlo@kmu.edu.tw; 6 Biostatistics Center, Kaohsiung Chang Gung Memorial Hospital, Kaohsiung 833, Taiwan; mary091234@gmail.com; 7 Department of Psychiatry, Kaohsiung Medical University Hospital, College of Medicine, Kaohsiung Medical University, Kaohsiung 807, Taiwan

**Keywords:** bipolar, depression, degenerative spine, psychiatric disorder, rehabilitation, surgery

## Abstract

The relationship between preexisting major psychiatric disorders and outcomes of spine surgery for degenerative thoracic/lumbar disease remains unclear. A 5% subset of inpatients was randomly selected from the Taiwan National Health Insurance Research Database. A total of 10,109 inpatients aged 18 years or over with degenerative thoracic/lumbar disease and underwent spine surgery met inclusion criteria. Major psychiatric disorders diagnosed by psychiatrists preceding index surgery, including anxiety disorder, depression disorder, bipolar disorder, schizophrenia and dementia, were identified. The prevalence of psychiatric disorders, and their differential risks on in-hospital and post-discharge outcomes were examined. 10.4% had major psychiatric disorders, of which depression (6.6%) and anxiety (4.9%) were most common. Logistic regression revealed increased risks of ventilator use in depression (OR = 1.62, 95% CI = 1.04–2.54, *p* < 0.05), extended hospitalization length in bipolar (OR = 1.77, 95% CI = 1.08–2.89, *p* < 0.05), and higher rehabilitation utilization in depression (OR = 1.25, 95% CI = 1.06–1.47, *p* < 0.01) and bipolar (OR = 1.69, 95% CI = 1.04–2.76, *p* < 0.05). Those patients with anxiety had a decreased risk of longer hospitalization duration (OR = 0.77, 95% CI = 0.60–0.98, *p* < 0.05), while those with dementia and schizophrenia had no change in risks. Preoperative recognition of major psychiatric disorders for risk and treatment assessment is suggested as people with preexisting depression or bipolar disorder have worse outcomes after spine surgery.

## 1. Introduction

As populations are ageing, low back pain has been recognized as the leading global health condition [1]. Almost 47% of individuals aged over 60 years are estimated to experience lumbar spinal stenosis [2]. The hospitalization rate and total number of surgical procedures for degenerative lumbar spine disease over the last decade have grown substantially [3,4]. As a result, a considerable demand for clinicians associated with the evaluation and treatment of degenerative thoracic or lumbar spine disease have emerged [4]. Therefore, further investigation that focuses on the risk factors of spinal surgery is clinically significant to assess and predict the treatment outcomes for individuals with degenerative thoracic or lumbar spine disease.

The prevalence of psychiatric disorders worldwide is expected to more than double by 2030 [5]. In Taiwan, the rate more than doubled in two decades, from 11.5% in 1990 to 23.8% in 2010 [6]. Patients with psychiatric disorders tend to be nonadherent to treatment because of poor reasoning and lack of insight regarding their illness and therapy. Moreover, preexisting psychiatric disorders may result in the delayed detection of physical diseases until an advanced stage, when prognosis is poor [7,8]. Mounting evidence suggests that anxiety or depression are associated with aging-associated physical illnesses and worsening treatment outcomes are increasing [9,10]. Moreover, the global incidence of dementia is rising as population aging accelerates [11]. Patients with dementia have poorer surgical outcomes and higher rates of physical comorbidities [12,13]. The impact of psychiatric disorders on treatment and care for various medical and surgical conditions is becoming more recognized. However, the prevalence of preoperative psychiatric disorders among patients with degenerative thoracic or lumbar spine disease remains poorly clarified. 

Preoperative psychological distress may affect disease outcome [14]. For instance, anxiety and depression are associated with back pain incidence [15] and its course of chronicity [16]. Patients with better mental health may experience more pain relief and improved physical function after spine surgery compared with those with poor mental health [17]. On the other hand, depression is associated with shorter survival time after cancer patients received operation [18]. Menendez et al. evaluated psychiatric disorders—namely depression, anxiety, schizophrenia, and dementia—in patients sampled from a national healthcare database who underwent major cervical, thoracic or lumbar spine surgery [19]. Diebo et al. also adopted a database research to examine depression, sleep, anxiety or stress disorder in patients with idiopathic scoliosis or degenerative disc disease [20]. The results suggest psychiatric disorders were associated with higher rates of physical comorbidities and were an independent risk factor for adverse events [19,20]. However, both studies included patients with diverse diagnoses of spinal disorders which escalates heterogeneity of study population, and thus limits explanations of degenerative spinal diseases in specific regions [19,20]. Besides, the studies lack information on how the psychiatric diseases were diagnosed or based by physicians or psychiatrists [20]. Moreover, the studies provide evidence mainly regarding depression and anxiety; therefore, leave gap between the association of other major psychiatric disorders (MPD), such as bipolar disorder and schizophrenia [21], with spinal surgery outcomes [22,23]. Because of the limitation of the study design, determining whether the negative effects are primarily due to preexisting major psychiatric disorders is difficult. Therefore, for accurate prediction of surgical prognosis for degenerative spine diseases in thoracic/lumbar region, a thorough investigation of the relationship of preoperative major psychiatric disorders based on psychiatrists and in-hospital and post-discharge outcomes of patients with degenerative thoracic or lumbar spine disease is warranted.

In view of this information, we conducted a nationwide study of inpatients elected from the National Health Insurance Research Database (NHIRD) to fill the above-mentioned gap with more comprehensive diagnoses of major psychiatric disorders before index hospitalization of spine surgery for degenerative thoracic or lumbar disease. We comprehensively evaluated the (1) prevalence of preoperative major psychiatric disorders which strictly diagnosed by psychiatrists, namely anxiety disorder, depression disorder, bipolar disorder, schizophrenia, and dementia, in patients with degenerative thoracic or lumbar spine disease undergoing surgery and (2) the impact of preoperative major psychiatric disorders on both in-hospital and post-discharge outcomes of index surgery.

## 2. Materials and Methods

### 2.1. Study Design and Setting

The study was approved by the Institutional Review Board of Chang Gung Memorial Hospital. The NHIRD, which is managed by the Bureau of National Health Insurance (NHI), consists of ambulatory claims database of Taiwanese. In Taiwan, the NHI program, which is universal and compulsory, was launched in 1995. Since 1996, more than 96% of Taiwan’s residents have joined the program. The Bureau handles reimbursements of medical expenses for hospital admissions or medical appointments and has access to patient medical records. These medical claims data are used in health science research and in the promotion of health care services [24]. 

The study used the subset of the NHIRD-TW, composed of one million enrollees selected randomly (1 out of 20 patients; 5%). The study population comprised patients aged 18 years or over who were diagnosed as having degenerative thoracic or lumbar spine disease and hospitalized for TLSS (*n* = 10,109) between 1998 to 2012 and followed until 2013. The primary diagnosis was based on the corresponding codes of the *International Classification of Diseases*, *Ninth Revision*, *Clinical Modification* (ICD-9-CM) for thoracic or lumbar disc displacement (722.1×, 722.5×, 722.72–722.73), thoracic or lumbar spondylosis (721.2–721.3, 721.4×), or other spinal stenosis (724.0×). The primary spine surgeries were classified by ICD-9-CM procedure codes for discectomy (80.51), laminectomy (03.09), and spinal fusion (81.0×).

### 2.2. Preoperative Major Psychiatric Disorders

The five primary preoperative major psychiatric disorders identified in the study population comprised anxiety disorder (293.84, 300.0×, 300.10, 300.2×, 300.3, 300.5, 300.89, 300.9, 308.×, 309.81), depression disorder (296.2, 296.3, 296.5, 296.6, 296.80, 296.82, 296.90, 298.0, 300.4, 309, 311), bipolar disorder (296.4–296.7, 296.0–296.16), schizophrenia (295–295.95), and dementia (290.×, 291, 294.10, 294.11). Anxiety, depression, bipolar and schizophrenia were strictly diagnosed by psychiatrists [25], and the diagnosis of dementia was allowed to be diagnosed by psychiatrists or neurologists. The physician specialties of psychiatrists and neurologists were defined in accordance with the record of NHI database. The date of first psychiatric diagnosis was prior to the index hospitalization. One patient may have more than one psychiatric disorder diagnosis. The 10,109 patients who underwent TLSS were further categorized into the control group without major psychiatric disorders (*n* = 9061, 89.6%) and the group with preoperative major psychiatric disorders (*n* = 1048, 10.4%). The group with major psychiatric disorders was further divided into subgroups of patients with the mentioned five disorders. Differences in demographic characteristics and surgical outcomes between the controls and group with major psychiatric disorders as well as each subgroup were examined. 

### 2.3. Physical Comorbidities

The diagnoses and ICD-9-CM codes of physical comorbidities were classified into hypertension (401–5.×), diabetes mellitus (250.×), dyslipidemia (272.0–4), chronic kidney disease (585.×), coronary heart disease 410–4.×), osteoporosis (733.0, 733.0×), chronic obstructive pulmonary disease (COPD) (490, 491.0–1, 491.20–2, 491.8–9, 492.0, 492.8494, 494.0–1, 496), cerebrovascular disease (CVD) (430–438), and fibromyalgia/myositis (729.1).

### 2.4. Surgical Outcomes: In-Hospitalization and Post-Discharge Outcomes

The in-hospital outcomes were index hospitalization duration and rates of ventilator use (procedure code: 57001B) during the index hospitalization. The post-discharge outcomes, including the rates of rehabilitation utilization and readmission for the same spine disease after the index hospitalization, to indicate accumulated utilization of health care relates with persistent functional deficits or surgical complications [26,27]. The continuous variable of index hospitalization stay was categorized into longer hospitalization lengths using the 75th percentile of hospitalization duration (11 days) as a cutoff point, except using the 25th percentile of hospitalization length (8 days) while comparing anxiety disorder with the controls due to relatively shorter duration.

### 2.5. Statistical Analysis

Data were analyzed using SPSS for Windows (version 21.0; SPSS, Chicago, IL, USA). Variables are presented as either means ± standard deviations or frequencies. 

Categorical and continuous variables were compared using the chi-square test and one-way analysis of variance to examine the effects among the overall psychiatric disorder group, five subgroups, and the control group. Multivariate logistic regression analysis was performed to examine the risks of each major psychiatric disorder in predicting the in-hospital outcomes including hospitalization stay, the need of ventilator use, and the post-discharge outcomes including rehabilitation utilization and readmission due to the same degenerative thoracic or lumbar spine disease after controlling for age, sex, spinal diagnosis and physical comorbidities. The odds ratios (OR) with 95% confidence intervals (95% CI) were calculated. All statistical tests were two-tailed and differences were considered to be significant at *p* < 0.05.

## 3. Results

Of the 10,109 patients analyzed, 1048 (10.4%) had preoperative major psychiatric disorders and 9061 (89.6%) did not (Table 1). Overall, the most common spinal diagnosis was spinal stenosis (50.0%), followed by disc displacement (44.1%) and spondylosis (5.9%). Discectomy (48.2%) was the most common procedure, followed by laminectomy (32.2%) and spinal fusion (19.6%). Patients with major psychiatric disorders were older (mean age: 59.5 ± 14.4 years vs. 57.1 ± 15.2 years, *p* < 0.001), predominantly female (62.1% vs. 49.9%, *p* < 0.001), and had higher rates of spinal stenosis diagnosis (55.4% vs. 49.4%, *p* < 0.001) and comorbidities with osteoporosis (29.0% vs. 25.6%, *p* = 0.019) and cerebrovascular disease (28.2% vs. 24.6%, *p* = 0.009). No significant between-group differences in surgical procedures or their numbers of such procedures were observed. 

Table 2 shows that patients with major psychiatric disorders had higher rates of ventilator use (3.7% vs. 2.2%, *p* = 0.003) during index hospitalization as well as a higher rate of rehabilitation utilization post discharge (51.5% vs. 46.3%, *p* = 0.001). We observed no significant between-group differences of hospitalization durations and readmission rate due to the same spinal diagnosis between patients with psychiatric disorders and the control group.

Among the participants, depression (6.6%) and anxiety (4.9%) were most common, followed by dementia (1.9%), bipolar (0.7%) and schizophrenia (0.4%). There was no age difference of anxiety, depression or bipolar comparing with controls. Schizophrenia and dementia were younger (mean age: 48.8 ± 15.1 years vs. 57.1 ± 15.2 years, *p* < 0.001) and older (mean age: 70.1 ± 10.9 years vs. 57.1 ± 15.2 years, *p* < 0.001). Anxiety (60.3% vs. 49.9%, *p* < 0.001), depression (67.0% vs. 49.9%, *p* < 0.001), or bipolar (69.1% vs. 49.9%, *p* = 0.002) were predominantly female, and bipolar (64.7% vs. 49.4%, *p* = 0.021) and dementia (68.5% vs. 49.4%, *p* < 0.001) had higher rates of spinal stenosis. Dementia had higher rates of spinal fusion surgery (29.9% vs. 19.3%, *p* < 0.001). Compared with controls, dementia had multiple physical comorbidities, depression had a high comorbidity with osteoporosis (29.7% vs. 25.6%, *p* = 0.022), and schizophrenia had a lower comorbidity with diabetes mellitus (18.4% vs. 36.4%, *p* = 0.022). Bipolar (11.1 ± 8.0 days vs. 9.3 ± 6.7 days, *p* = 0.029) had longer hospitalization stays with no difference of in-hospital outcomes. Dementia required extended hospitalization stays (11.0 ± 8.6 days vs. 9.3 ± 6.7 days, *p* < 0.001). Ventilator use rates were higher in patients with depression (3.4% vs. 2.2%, *p* = 0.043) and dementia (5.6% vs. 2.2%, *p* = 0.002). Post-discharge rehabilitation utilization rates were higher in patients with depression (52.5% vs. 46.3%, *p* = 0.002) and bipolar disorder (60.3% vs. 46.3%, *p* = 0.022). Schizophrenia had no difference of in-hospital or post-discharge outcomes (Table 3).

Multivariate logistic regression analysis (Table 4) reveals that patients with anxiety had a decreased risk of hospitalization stays longer than 8 days (OR = 0.77, 95% CI = 0.60 to 0.98, *p* = 0.035). Patients with depression had an increased risk of ventilator use (OR = 1.62, 95% CI = 1.04 to 2.54, *p* = 0.033) during index hospitalization. Patients with bipolar disorder had an increased risk of hospitalization stays longer than 11 days (OR = 1.77, 95% CI = 1.08 to 2.89, *p* = 0.024). Both depression and bipolar disorder had an increased risk of rehabilitation utilization rates after discharge (OR = 1.25, 95% CI = 1.06 to 1.47, *p* = 0.007 for depression; OR = 1.69, 5% CI = 1.04 to 2.76, *p* = 0.035 for bipolar). We observed no statistical differences of risks in surgical outcomes among patients with schizophrenia and dementia in comparison with the controls.

## 4. Discussion

To our knowledge, this work is the first study to investigate the prevalence of major psychiatric disorders diagnosed based on psychiatrists with more comprehensive spectrum prior to the index surgery and their relationship with postoperative in-hospital and post-discharge outcomes among patients with degenerative thoracic or lumbar spine disease. The principal findings are as follows: (1) depression and anxiety were the most prevalent, followed by dementia, bipolar disorder, and schizophrenia; (2) mood disorder, including depression and bipolar disorder, was an independent risk factor of in-hospital and post-discharge adverse events; (3) anxiety disorder had a decreased risk of extended hospitalization stays.

In our work, the prevalence of major psychiatric disorders (10.4%) is lower than that in the general population (30%) [28]. The overall prevalence of depression (6.6%) and bipolar disorder (0.7%) is lower than that in the general population (8.2% and 1.6–1.8%, respectively) [28,29]. The prevalence of anxiety (4.9%) is much lower than that in the general population (18.1%) [28] and patients with type 2 diabetes (11.0–13.7%) [30]. One study [19] of patients undergoing major spine surgery reported a lower prevalence of anxiety (2.5%) and schizophrenia (0.2%) than our results. The prevalence of dementia (1.9%) between the two studies is comparable, but both rates are lower than that of the general population, which ranges from 12.2% to 16.6% [31]. On the contrary, another study reported high prevalence of depression (59%) and anxiety (24%) disorders among patients receiving surgery for adult spinal deformity [20]. The distribution of the ranking major psychiatric disorders in our study, said depression, anxiety and dementia, is in agreement with the study which examined psychiatric diagnoses in elective orthopedic surgery, though the information of specific psychiatric disorders for lumbar spine surgery, such as bipolar disorder and dementia, is lacking for comparison [32,33]. This discrepancy may be explained by the diversity of sample populations and variations in both the diagnostic criteria and the specialists who made the diagnoses. Nevertheless, our result suggests that the prevalence of major psychiatric disorders among patients with degenerative thoracic or lumbar spine disease requiring surgical intervention is underestimated, perhaps because of the underdetection of psychiatric comorbidities or the relatively lower surgical rate in patients with preexisting psychiatric disorders [34]. Clinicians are advised to be aware of patients’ mental state in evaluating them for spine surgery.

Regarding the mood disorders of depression and bipolar disorder, although patients with bipolar disorder were younger and had fewer physical comorbidities than those with dementia, they had higher rates of spinal stenosis and 1.77-fold higher odds of extended hospitalization stays compared with the controls. On the other hand, patients with depression had 1.62-fold higher odds of ventilator use after index surgery for degenerative spine disease compared with the controls. This finding is in agreement with previous study which suggests that patients with depression demonstrate ventilation insufficiency compared with controls [35]. Among depression patients, such augmented efforts on ventilation efficiency may be contributed by the altered autonomic function sensitivity and an early onset of lactic acidosis, while the correlation with physical inactivity is nonsignificant [35]. Patients with depression, if being comorbid with COPD, exhibit increased risks of moderate-severe exacerbations [36]. Taken together, it would be important to recommend a program with spirometry prior to spine surgery to look into relation of pre-surgery lung parameters to the post-surgery respiratory complications. 

Moreover, both bipolar disorder and depression demonstrated an independent risk factor of post-discharge rehabilitation utilization in our study, which may indicate their reduced physical outcomes such as persistent pain or neurological deficits. Despite their readmission rates were similar to that of controls, our finding reflects the relatively poorer surgical outcomes among patients with mood disorders, whether during index hospitalization or after discharge. The phenomenon may be partially ascribed to the fact that patients with bipolar disorder present with the most severe clinical symptoms among patients with mood disorders [28]. In comparison with patients with depression, their mood instability may negatively affect their treatment-seeking behavior and comprehension and communication with clinicians [22,37]. In addition, clinicians may attribute these patients’ symptoms to their psychiatric disorder rather than physical discomfort [38]. Thus, in patients with bipolar disorder, spine conditions may tend to be diagnosed at later stages, after the development of neurological deficits; moreover, the time window between the definite spinal diagnosis and surgical admission is longer than that in patients with depression [39].

In addition, the level of physical activity has been shown to be an important predictor for the surgery prognosis [40]. However, the relationship between psychiatric disorders and physical activity is inconsistent. One study found variations in physical activity across psychiatric disorders [41]; however, no association is shown in a longitudinal study [42]. Further investigations are warranted to examine the pre-operative physical activity in patients with psychiatric disorders and its association with surgical outcomes. Above all, early diagnosis and disease-specific treatment strategies are suggested to facilitate the preventive interventions of risk reduction in high-risk groups [43], especially for patients with preoperative depression or bipolar disorder. 

Compared with the controls, patients with anxiety disorder had 0.77-fold lower odds of extended hospitalization stays without significant differences of in-hospital or post-discharge outcomes. Although previous studies found that among patients with degenerative thoracic or lumbar spine disease, neither preoperative depression nor anxiety is correlated with improvement of postoperative outcomes such as life quality, physical functioning, or perceived pain [44,45]. The finding may hint that patients with anxiety mood disorder had favorable surgical outcomes than those with mood disorder. The phenomenon may be explained by the fact that the patients with anxiety use healthcare services more frequently [46] and their diagnoses of degenerative thoracic or lumbar spine disease may be made earlier, before the condition of spinal disease has progressed to advanced stages. In our work, hypochondriasis was not included in the anxiety disorder due to reported small sample size (0.01%) [25]. However, the qualification for surgery may depend on the patients’ hypochondriacal symptoms. Further study by focusing on the hypochondriasis in patients with anxiety or depression is important to evaluate their impact on spine surgery outcomes. By contrast, patients with schizophrenia were almost 8 years younger than controls when they underwent TLSS. This finding is in agreement with previous study [19]. The result may suggest that patients with schizophrenia may present with degenerative spine conditions at younger ages because of the accelerated aging characteristic of such diseases [47]. Moreover, the result of no differences in either in-hospital or post-discharge complications is in part supported by the finding of relatively decreased risks of physical complications in patients with regular antipsychotic adherence [48]. Further longitudinal studies assessing the clinical course and treatment adherence of psychiatric disorders before and after surgery are necessary to evaluate their impact on both short- and long-term outcomes.

In the present study, although dementia demonstrated poorest in-hospital outcomes, including prolonged hospitalization and higher risks of ventilator use, there was no increased risk of such surgical outcomes after controlling for confounding factors. Evidence on the associations of age and surgical outcomes has been inconsistent. Some studies have not identified advanced age to be a predictor of surgical outcomes [49]. However, others have reported correlations of advanced age with higher rates of surgical complications and mortality [50,51]. Menendez et al. also found that patients with dementia have the worst in-hospital mortality after spine surgery [19]. This inconsistency may be due to the different outcome measures and the fact that whether more severe physical comorbidities or the diagnosis of dementia contributes to higher risks of poorer surgical outcomes [12,13]. Nevertheless, clinicians should take particular care in evaluating patients with preexisting dementia who are associated with multiple physical comorbidities and design individualized treatment strategies to minimize in-hospital complications from surgical interventions for degenerative thoracic or lumbar spine disease. 

This study has several strengths. First, the nationwide population-based database provides prevalence of specific major psychiatric disorders diagnosed strictly by psychiatrists and reduces the bias regarding internal validity of psychiatric diagnosis between non-psychiatrists and psychiatrists [52]. Second, we expanded the spectrum of psychiatric disorders, including bipolar disorder and schizophrenia, and gave more comprehensive information of the differential outcome risks of each psychiatric disorder among patients with degenerative spine disease to fill the gap of previous studies in accordance with ageing population. Furthermore, the findings of both in-hospital and post-discharge outcomes during index surgery offer clinicians related evidence for managing healthcare strategy in patients with degenerative thoracic or lumbar spine disease. 

The following limitations must be addressed. First, this work suggests a temporal correlation between preoperative psychiatric disorders and the outcomes for patients undergoing surgery, but confounding factors including active/remitted status of psychiatric disorders explaining this potentially causal relationship cannot be ruled out. Further prospective studies are warranted to investigate the effect of clinical course and prognosis of psychiatric disorders on surgical outcomes. Second, this work mainly adopted the primary spine procedure code and could not provide data regarding dual procedures. Third, in this study, we captured readmissions due to the same spinal diagnoses, but did not include the conditions of readmissions due to possible complications of the spinal index surgery. Therefore, the result of readmission rate could not generalize to the whole population of degenerative thoracic or lumbar disease underwent spine surgeries. Moreover, we did not evaluate complications, more granular post-discharge outcomes and mortality. The results of this study could not provide information to reflect overall healthcare utilization and quality, and therefore merit further investigation. Fourth, the severity of degenerative thoracic or lumbar spine disease before and after surgery and its relationship with the surgical outcomes necessitates further evaluation. Finally, because we could not confirm whether discharge status referred to nonroutine discharge, interpretations of hospitalization duration in the present study should be made with caution. 

## 5. Conclusions

Preoperative major psychiatric disorders demonstrated a heterogeneity of risks of thoracic or lumbar spine surgery outcomes. Depression and bipolar disorders are independent risk factors of poorer surgical outcomes among major psychiatric disorders. Clinicians are advised to identify major psychiatric disorders for risk assessment and treatment strategy evaluation in patients undergoing surgery for degenerative thoracic or lumbar spine disease. Longitudinal studies are warranted to determine the definite diagnosis and treatment adherence of psychiatric disorders before and after surgery to examine their impact on the outcomes, including complications, more detailed post-charge events and mortality.

## Figures and Tables

**Table 1:** Comparison of demographic data and physical comorbidities between the control group and patients with major psychiatric disorders who underwent thoracic or lumbar spine surgery.

	Control (*n* = 9061)	Major Psychiatric Disorders (*n* = 1048)	*p*
Demographic data			
Age	57.1 ± 15.2	59.5 ± 14.4	<0.001 ***
Female, *n* (%)	4518 (49.9)	651 (62.1)	<0.001 ***
Diagnosis, *n* (%)			<0.001 ***
Disc displacement	4050 (44.7)	409 (39.0)	
Spondylosis	538 (5.9)	58 (5.5)	
Spinal stenosis	4473 (49.4)	581 (55.4)	
Spine procedure, *n* (%)			0.193
Discectomy	4387 (48.4)	489 (46.7)	
Laminectomy	2922 (32.3)	332 (31.7)	
Spine fusion	1752 (19.3)	227 (21.6)	
Number of spine procedure			0.738
1	7575 (83.7)	871 (83.3)	
≧2	1478 (16.3)	175 (16.7)	
Urbanization, *n* (%)			0.906
Urban	4911 (54.2)	566 (54.0)	
Rural	4150 (45.8)	482 (46.0)	
Physical comorbidities, *n* (%)			
Hypertension	5624 (62.1)	667 (63.6)	0.319
Diabetes	3294 (36.4)	383 (36.5)	0.903
Hyperlipidemia	3957 (43.7)	489 (46.7)	0.065
Chronic kidney disease	895 (9.9)	105 (10.0)	0.884
Coronary heart disease	3153 (34.8)	369 (35.2)	0.791
Osteoporosis	2324 (25.6)	304 (29.0)	0.019 *
COPD	3055 (33.7)	353 (33.7)	0.983
Cerebrovascular disease	2227 (24.6)	296 (28.2)	0.009 **
Fibromyalgia/myositis	4415 (48.7)	506 (48.3)	0.786

COPD, chronic obstructive pulmonary disease; * *p* < 0.05, ** *p* < 0.01, *** *p* < 0.001.

**Table 2:** Comparison of in-hospital and post-discharge outcomes between the control group and patients with major psychiatric disorders who underwent thoracic or lumbar spine surgery.

	Control (*n* = 9061)	Major Psychiatric Disorders (*n* = 1048)	*p*
In-hospital outcomes			
Hospitalization stay, days	9.3 ± 6.7	9.6 ± 6.7	0.242
Ventilator use, *n* (%)	202 (2.2)	39 (3.7)	0.003 **
Post-discharge outcomes			
Rehabilitation, *n* (%)	4199 (46.3)	540 (51.5)	0.001 **
Readmission, *n* (%)	1185 (13.1)	129 (12.3)	0.483

Readmission, readmission due to the same spinal diagnosis. ** *p* < 0.01.

**Table 3:** Comparison of demographic data, physical comorbidities, in-hospital and post-discharge outcomes between the control group and subgroups. of patients with major psychiatric disorders who underwent thoracic or lumbar spine surgery.

	Major Psychiatric Disorders									
	Anxiety	*p*	Depression	*p*	Bipolar	*p*	Schizophrenia	*p*	Dementia	*p*
Total number (%)	494 (4.9)		667 (6.6)		68 (0.7)		38 (0.4)		197 (1.9)	
Demographic data										
Age, years	57.6 ± 14.3	0.426	58.1 ± 13.7	0.063	57.4 ± 14.4	0.843	48.8 ± 15.1	<0.001 ***	70.1 ± 10.9	<0.001 ***
Female, *n* (%)	298 (60.3)	<0.001 ***	447 (67.0)	<0.001 ***	47 (69.1)	0.002 **	18 (47.4)	0.759	111 (56.3)	0.072
Diagnosis, *n* (%)		0.062		0.057		0.021 *		0.244		<0.001 ***
Disc displacement	203 (41.1)		268 (40.2)		19 (27.9)		20 (52.6)		53 (26.9)	
Spondylosis	22 (4.5)		38 (5.7)		5 (7.4)		0 (0.0)		9 (4.6)	
Spinal stenosis	269 (54.5)		361 (54.1)		44 (64.7)		18 (47.4)		135 (68.5)	
Spine procedure, *n* (%)		0.173		0.559		0.224		0.247		<0.001 ***
Discectomy	257 (52.0)		313 (46.9)		26 (38.2)		23 (60.5)		76 (38.6)	
Laminectomy	140 (28.3)		214 (32.1)		25 (36.8)		11 (28.9)		62 (31.5)	
Spinal fusion	97 (19.6)		140 (21.0)		17 (25.0)		4 (10.5)		59 (29.9)	
Physical comorbidities, *n* (%)										
Hypertension	301 (60.9)	0.612	409 (61.3)	0.701	42 (61.8)	0.959	19 (50.0)	0.126	150 (76.1)	<0.001 ***
Diabetes mellitus	167 (33.8)	0.251	230 (34.5)	0.332	26 (38.2)	0.748	7 (18.4)	0.022 *	89 (45.2)	0.011 *
Hyperlipidemia	235 (47.6)	0.089	316 (47.4)	0.063	29 (42.6)	0.865	11 (28.9)	0.068	84 (42.6)	0.773
Chronic kidney disease	40 (8.1)	0.195	60 (9.0)	0.460	8 (11.8)	0.604	1 (2.6)	0.174	33 (16.8)	0.001 **
Coronary heart disease	160 (32.4)	0.273	230 (34.5)	0.869	23 (33.8)	0.867	9 (23.7)	0.151	77 (39.1)	0.211
Osteoporosis	129 (26.1)	0.818	198 (29.7)	0.022 *	20 (29.4)	0.479	9 (23.7)	0.782	75 (38.1)	<0.001 ***
COPD	159 (32.2)	0.484	216 (32.4)	0.482	22 (32.4)	0.813	10 (26.3)	0.335	80 (40.6)	0.043 *
Cerebrovascular disease	118 (23.9)	0.728	181 (27.1)	0.139	12 (17.6)	0.186	5 (13.2)	0.103	92 (46.7)	<0.001 ***
Fibromyalgia/myositis	229 (46.4)	0.305	349 (52.3)	0.073	38 (55.9)	0.239	16 (42.1)	0.415	78 (39.6)	0.011 *
In-hospital outcomes										
Hospitalization stay, days	8.8 ± 5.3	0.033 *	9.5 ± 6.4	0.518	11.1 ± 8.0	0.029 *	9.7 ± 7.4	0.714	11.0 ± 8.6	<0.001 ***
Ventilator use, *n* (%)	16 (3.2)	0.143	23 (3.4)	0.043 *	3 (4.4)	0.196	1 (2.6)	0.577	11 (5.6)	0.002 **
Post-discharge outcomes										
Rehabilitation, *n* (%)	245 (49.6)	0.158	350 (52.5)	0.002 **	41 (60.3)	0.022 *	22 (57.9)	0.154	95 (48.2)	0.600
Readmission, *n* (%)	58 (11.7)	0.390	92 (13.8)	0.598	10 (14.7)	0.692	6 (15.8)	0.621	19 (9.6)	0.156

COPD, chronic obstructive pulmonary disease; Readmission, readmission due to the same spinal diagnosis; * *p* < 0.05, ** *p* < 0.01, *** *p* < 0.001.

**Table 4:** Logistic regression analysis of in-hospital and post-discharge outcomes between the control group and subgroups of patients with major psychiatric disorders who underwent thoracic or lumbar spine surgery.

Variables	Anxiety		Depression		Bipolar		Schizophrenia		Dementia	
	OR 95% CI	*p*	OR 95% CI	*p*	OR 95% CI	*p*	OR 95% CI	*p*	OR 95% CI	*p*
Patients vs. controls										
In-hospital outcomes										
Hospitalization stay ^a^	0.77 (0.60, 0.98)	0.035 *	1.02 (0.87, 1.20)	0.798	1.77 (1.08, 2.89)	0.024 *	1.77 (0.92, 3.41)	0.088	1.05 (0.78, 1.40)	0.759
Ventilator use	1.51 (0.89, 2.55)	0.125	1.62 (1.04, 2.54)	0.033 *	2.13 (0.65, 6.98)	0.214	2.07 (0.27, 15.80)	0.482	1.32 (0.70, 2.51)	0.391
Post-discharge outcomes										
Rehabilitation	1.12 (0.93, 1.34)	0.220	1.25 (1.06, 1.47)	0.007 **	1.69 (1.04, 2.76)	0.035 *	1.78 (0.93, 3.40)	0.083	1.02 (0.75, 1.38)	0.914
Readmission	0.91 (0.69, 1.21)	0.513	1.12 (0.89, 1.41)	0.334	1.20 (0.61, 2.36)	0.593	1.27 (0.53, 3.05)	0.598	0.78 (0.48, 1.26)	0.309

Readmission, readmission due to the same spinal diagnosis. OR, Odds ratio; 95% CI, 95% Confidence interval. * *p* < 0.05, ** *p* < 0.01. Adjusted for age, sex, degenerative spine disease, physical comorbidities. ^a^ Cutoff points of hospitalization stays was 11 days in subgroups of major psychiatric disorder, except 8 days for anxiety disorders.

## Data Availability

Restrictions apply to the data availability. Data was obtained from the National Health Insurance database and is not available to the authors if permission is not granted by the National Health Insurance Administration of Taiwan.

## Abbreviations

BNHI: Bureau of National Health Insurance; ICD-9-CM, International Classification of Diseases Ninth Revision, Clinical Modification; OR, Odds ratios; NHI, National Health Insurance; NHIRD-TW, National Health Insurance Research Database of Taiwan.
